# Mitoplastomic discordance in Brassicaceae phylogenomics confirms the complex evolutionary history of the family

**DOI:** 10.1093/aob/mcaf084

**Published:** 2025-05-20

**Authors:** Lisa J M A Dominicus, Ihsan A Al-Shehbaz, Dmitry A German, Klaus Mummenhoff, Nikolai M Hay, Martin A Lysák, Marcus A Koch, Frederic Lens, Kasper P Hendriks

**Affiliations:** Functional Traits, Naturalis Biodiversity Center, Leiden, The Netherlands; Missouri Botanical Garden, St. Louis, MO, USA; South-Siberian Botanical Garden, Altai State University, Barnaul, Russia; Department of Biology, Botany, University of Osnabrück, Osnabrück, Germany; Department of Biology, Duke University, Durham, NC 27708, USA; CEITEC–Central European Institute of Technology, Masaryk University, Kamenice 5, Brno 625 00, Czech Republic; Centre for Organismal Studies (COS), Heidelberg University, Im Neuenheimer Feld 345, 69120 Heidelberg, Germany; Functional Traits, Naturalis Biodiversity Center, Leiden, The Netherlands; Plant Sciences, Institute of Biology Leiden, Leiden University, Leiden, The Netherlands; Functional Traits, Naturalis Biodiversity Center, Leiden, The Netherlands; Department of Biology, Botany, University of Osnabrück, Osnabrück, Germany

**Keywords:** Brassicaceae, Cruciferae, mustard family, phylogenomics, mitogenomics, cytonuclear discordance, mitoplastomic discordance, rogue taxa

## Abstract

**Background and Aims:**

The phylogeny of the Brassicaceae family has traditionally been inferred from plastid and nuclear DNA. However, early studies were limited by the availability of genetic markers and incomplete taxon sampling. Recent phylogenomic studies, leveraging more densely sampled nuclear and plastid datasets, have resolved many taxonomic uncertainties. These studies either targeted complete plastomes or provided extensive representation of the nuclear genome. Nevertheless, substantial cytonuclear discordance, poorly resolved backbone relationships and challenges in placing ‘rogue taxa’ have left unresolved questions about deeper relationships, notably of the five supertribes of the family. In this context, we performed the first phylogenomic analysis of the slower-evolving, maternally inherited mitogenome, which presents a promising avenue for resolving deeper phylogenetic nodes.

**Methods:**

Using published mitogenomes from nine Brassicaceae species, we generated a mitogenomic reference file to recover mitogenomic sequencing read data from Hendriks *et al*. (2023. Global Brassicaceae phylogeny based on filtering of 1000-gene dataset. *Current Biology: CB*  **33**: 4052–4068.e6). Subsequently, we reconstructed a codon-aware mitogenomic supermatrix, alongside updated nuclear (281 genes) and plastome (76 genes) supermatrices and inferred family-wide maximum likelihood phylogenies from each of these three genomes. Congruence among the resulting phylogenies was assessed thoroughly.

**Key Results:**

We present the first densely sampled family-wide mitogenomic Brassicaceae phylogeny, including 167 species, 145 genera (40% of the family) and 40 tribes (69% of the family), and the first family-wide phylogenomic comparison based on all three plant genomes. Although cytonuclear discordance was evident, we also uncovered strong phylogenomic discordance between the two organellar genomes (mitogenome and plastome), coined here as ‘mitoplastomic discordance’. Our findings offer new insights into the placement of several rogue and previously unplaced taxa.

**Conclusions:**

Phylogenomic discordance in Brassicaceae was more pervasive than expected. Although bifurcating phylogenies offer clear evolutionary hypotheses, they do not fully capture evolutionary complexities. Our results have implications for understanding Brassicaceae evolution, taxonomy and systematics, shedding light on processes such as hybridization and genome duplication, commonly resulting in evolutionary reticulation.

## INTRODUCTION

The Brassicaceae Tree of Life (BrassiToL) has traditionally been inferred from plastid (Beilstein *et al.*, 2010; Liu *et al.*, 2011, 2021; Guo *et al.*, 2017) or nuclear (Bailey *et al.*, 2006; Beilstein *et al.*, 2008; German *et al.*, 2009; Warwick *et al.*, 2010; Liu *et al.*, 2011, 2021; Huang *et al.*, 2016; Nikolov *et al.*, 2019) DNA sequences. Until recently, such phylogenies were hampered by limited marker sampling (generally fewer than five markers) and/or limited taxon sampling. The first issue was tackled by Nikolov *et al.* (2019), who designed a 764 single-copy nuclear gene set to allow accurate inference of the nuclear Brassicaceae phylogeny. The second issue was tackled by Hendriks *et al.* (2023),; https://treeoflife.naturalis.nl/brassicaceae) who generated the most densely sampled nuclear and plastid Brassicaceae phylogenies to date, including representatives of 92% of the genera and all 58 tribes, based on the analysis of 1081 nuclear and 76 plastid genes, respectively. Together, their work resolved long-standing issues regarding the position of many tribes and genera and laid the foundation for describing several new tribes (Arabidopsideae, Asperuginoideae, Hemilophieae and Schrenkielleae) and supertribes (Arabodae, Camelinodae, Heliophilodae and Hesperodae) and the formal description of subfamily Aethionemoideae as opposed to Brassicoideae (German *et al.*, 2023; Givnish, 2023). Despite these substantial advancements, the relationships among many deeper nodes (the ‘family backbone’) and the placement of various so-called ‘rogue taxa’ remain unresolved. These rogue taxa (defined as those that shift positions in the phylogeny depending on the subset of data or methodology used) include tribes such as Anastaticeae, Biscutelleae, Cochlearieae, Iberideae and Megacarpaeeae (Walden *et al.*, 2020; German *et al.*, 2023; Hendriks *et al.*, 2023).

Many factors have been suggested to contribute to the challenges in clarifying the (deeper) phylogenetic relationships within the Brassicaceae family. First, artefacts might introduce errors, such as substitution saturation or incorrect phylogenetic inference owing to compositional heterogeneity (differences in the nucleotide composition attributable to mutational bias or evolutionary rate variation, for example; Liu *et al.*, 2014). Second, biological factors such as chloroplast capture (Kawabe *et al.*, 2018), polyploidy resulting from whole-genome duplication or hybridization (Franzke *et al.*, 2011; Huang *et al.*, 2020), incomplete lineage sorting and introgression can lead to phylogenetic incongruences. Polyploidy is well known to be present widely in the Brassicaceae (Mandáková *et al.*, 2017*a*), triggering rapid radiation (Schranz *et al.*, 2012), adding a further challenge in phylogenetic reconstructions (Hohmann *et al.*, 2015; Huang *et al.*, 2020).

A promising addition for resolving the deep-time Brassicaceae relationships might come from the mitochondrial genome, which has not been studied thoroughly in Brassicaceae at the family level before. First, the mitogenomic mutation rate is much lower than that of either the plastome or nuclear genome (Wolfe *et al.*, 1987; Palmer and Herbon, 1988), with rates in seed plants at a ratio of 1:3:10, respectively (Drouin *et al.*, 2008), potentially mitigating the risk of substitution saturation. Second, mitochondria, like chloroplasts (Palmer, 1985; Daniell *et al.*, 2016), are predominantly inherited maternally, with only rare cases of biparental (Corriveau and Coleman, 1988; Duminil, 2014) or paternal inheritance (Duminil, 2014; Shen *et al.*, 2015; Chybicki *et al.*, 2016), potentially alleviating the challenge of a distorted allopolyploid signal (albeit at the cost of a partial loss of parental information). Third, although mitochondrial genomes in plants vary widely in size (often 200–400 kb, with the largest flowering plant mitogenome recorded being 11 300 kb in *Silene conica*, Caryophyllaceae; Sloan *et al.*, 2012), and the structural complexity of the mitogenome evolves rapidly (Palmer and Herbon, 1988; Sloan *et al.*, 2012), the number of mitogenomic genes remained relatively constant (Palmer *et al.*, 2000).

Using the slower-evolving mitogenome to resolve deeper evolutionary relationships in land plants is not a novel concept, yet its inclusion in phylogenetic analyses remains relatively uncommon. Beyond the Brassicaceae, several phylogenetic studies have specifically targeted the mitogenome, e.g. focusing on land plants (Liu *et al.*, 2014; Sousa *et al.*, 2020), angiosperms (Qiu *et al.*, 2000; Xue *et al.*, 2022), mycoheterotrophic lineages (Lin *et al.*, 2022), order Pandanales (Soto Gomez *et al.*, 2020), the Rubiaceae family (Rydin *et al.*, 2017), genus *Pelargonium* (Bakker *et al.*, 2000) and genus *Potentilla* (Xue *et al.*, 2024). Many of these studies have identified incongruences between phylogenies derived from mitogenomes and plastomes. Nonetheless, mitochondrial genomes have frequently demonstrated their value in resolving deep evolutionary relationships (Van de Paer *et al.*, 2016; Lin *et al.*, 2022; Xue *et al.*, 2022).

Mitogenomic phylogenetic studies within the Brassicaceae have focused primarily on single genes. Franzke *et al.* (2009) analysed the NADH subunit 4 gene in 49 Brassicaceae species spanning much of the diversity of the family, but could not resolve most deep polytomies. Likewise, Couvreur *et al.* (2010) examined the *nad4* intron 1 and integrated these data with nuclear and plastome gene sequences in a supermatrix analysis of 226 Brassicaceae species. Despite the extended dataset, deeper branches remained poorly supported, probably owing to cytonuclear discordance (i.e. evolutionary differences between the nuclear genome and cytoplasmic genomes, such as the plastome and mitogenome). Likewise, Liu *et al.* (2011) investigated the *nad7* intron 2 in 71 Chinese Brassicaceae taxa, but their results also showed relatively low support for backbone nodes and poor resolution of more recent intertribal clades. Interestingly, both Franzke *et al.* (2009) and Liu *et al.* (2011) found discrepancies between mitochondrial and plastid-derived phylogenies in Brassicaceae. Qiao *et al.* (2020) presented a comparison between plastome and mitogenomic phylogenies (with a focus towards genus *Brassica*), but the first comparative phylogenomic analysis comparing all three plant genomes (nuclear, plastid and mitochondrial) in Brassicaceae was applied to unravel the reticulate evolutionary history of tribe Cochleariae at the species level (Wolf *et al.*, 2021). However, these studies do not entail the complete family.

To elucidate the deeper relationships within the entire Brassicaceae, we reconstructed the most comprehensive and robust mitogenomic family phylogeny to date. We compared it with the nuclear and plastome phylogenies. To this end, we reanalysed raw sequencing data from the study by Hendriks *et al.* (2023), used an improved codon-aware gene alignment strategy and applied a uniform phylogenomic approach to each of the three genomic datasets for fair comparison.

## MATERIALS AND METHODS

### Species sampling and the mitogenome

We filtered all mitogenomic DNA reads from the raw sequencing data (target capture sequencing with genome spiking) of Hendriks *et al.* (2023), available from NCBI SRA *BioProject PRJNA806513* (340 samples; Supplementary Data Table S1). Like Hendriks *et al.* (2023), we added a subset of data from Nikolov *et al.* (2019; 37 samples; Supplementary Data Table S2; *BioProject PRJNA518905*). To filter out the mitogenomic reads, we created a new Brassicaceae-specific mitogenomic target genes file to map raw reads. This included sequence data from nine previously published and well-annotated mitogenomes (Supplementary Data Table S3) covering most of the taxonomic diversity of the family (supertribes Arabodae, Brassicodae and Camelinodae). The exons of 32 protein-coding genes, as defined for *Arabidopsis thaliana* (Unseld *et al.*, 1997) were selected from each mitogenome, and exons were concatenated by gene; 24 ribosomal RNA and transfer RNA genes were excluded, because they were either very short (<100 bp) or not consistently annotated across the reference mitogenomes (Supplementary Data Table S4). In cases where two exon copies were annotated in a mitogenome, we ignored the most dissimilar copy, most probably representing a paralogue (Supplementary Data Table S4). Finally, trailing stop codons were removed (Supplementary Data Table S5). Likewise, we used an unpublished reference file for 76 plastid genes for the plastome, based on 15 previously published chloroplast genomes from across the Brassicaceae (Supplementary Data Table S6; Hay *et al.*, 2023).

#### Sequence mapping and gene alignments.

We used the bioinformatic pipeline of Hendriks *et al.* (2023) in a parallel computing environment using GNU Parallel (Tange, 2011), with the following improvements. For mapping raw sequencing data, we turned to HybPiper v.2.1.2 (Johnson *et al.*, 2016) instead of the original version, v.1.3.1. More importantly, we applied the codon-aware alignment algorithm of OMM-MACSE (Ranwez *et al.*, 2018), which greatly outperforms (in terms of alignment quality) alignment tools ignoring codon-position information. To be methodologically consistent when comparing species phylogenies from all three genomes (i.e. mitogenome, nuclear genome and plastome) and because our methods differed slightly from those of Hendriks *et al.* (2023), we reanalysed the same raw sequencing data using reference genomes for the nuclear genome (Supplementary Data Table S7) and plastome (Supplementary Data Table S8). For the nuclear genome, we used the gene reference file from the ‘mixed baits’ approach of Hendriks *et al.* (2021), designed for combined target capture sequencing of 764 Brassicaceae-specific genes (Nikolov *et al.*, 2019) and 353 Angiosperm-wide genes (Johnson *et al.*, 2019); a method successfully applied in several recent phylogenomic studies in Brassicaceae (Farhat *et al.*, 2023; Hay *et al.*, 2023; Hendriks *et al.*, 2023; Walden *et al.*, 2024).

#### Genetic variation.

We focused on the mitogenome as a potential means to resolve the early evolutionary history of the Brassicaceae, because genetic variation is commonly much lower in the mitogenome than in the nuclear genome and plastome. To test explicitly for this in the Brassicaceae, we calculated genetic mean pairwise distance (MPD) for each gene from each of the three genomes studied using the function ‘dist.dna’ from R-package ‘ape’ (Paradis and Schliep, 2019).

#### Gene sampling.

Not all 32 mitogenomic genes could be recovered for all samples, because the target capture sequencing approach of Hendriks *et al.* (2023) introduced a heavy bias towards selecting the 1081 nuclear genes. To allow assessment of the impact of incomplete gene sampling, we defined three gene sampling strategies to reconstruct three mitogenomic phylogenies (Table 1). In the ‘relaxed’ strategy, we aimed to keep as many samples (and therefore species, genera and tribes) as possible, setting a lower threshold of five genes per sample; any samples with fewer than five genes were excluded before phylogenetic reconstruction. The ‘selective’ (minimum of 15 genes per sample) and ‘stringent’ (minimum of 25 genes per sample) strategies were aimed at including more genes per sample, hence more genetic information, but at the cost of species sampling. Because plastid genes were on average more informative (more variation per gene) than the mitogenomic genes (see Results), we applied a single lower threshold of five genes per sample (Supplementary Data Table S9). Finally, we focused on the 297 nuclear genes identified by Hendriks *et al.* (2023) for the nuclear genome in their so-called ‘strict’ routine. This strategy represents a subset of the 1081 nuclear genes studied, refined by excluding all genes with a single nucleotide polymorphism fraction of >0.02 across all samples. Using our improved alignment strategy, 281 of the 297 genes passed our bioinformatic pipeline (Supplementary Data Table S10). All analyses had representatives from all supertribes, plus tribe Aethionemeae.

**Table 1.** Brassicaceae phylogenomic sampling overview. The focus in descriptions and comparisons is on the datasets in bold.

Genome, sampling strategy	Threshold minimum number of genes per sample	Number of genes studied	Species	Genera	Tribes
Mitochondrial, relaxed	5	32	248	214	49
**Mitochondrial,** **selective**	**15**	**32**	**167**	**145**	**40**
Mitochondrial, stringent	25	32	115	101	35
**Plastome**	**5**	**76**	**338**	**291**	**57**
**Nuclear genome**	**–**	**281**	**360**	**311**	**57**

#### Phylogenetic reconstructions.

The mitogenome and plastome are generally inherited as single non-recombining units; therefore, their phylogenies can best be inferred using a supermatrix approach. We reconstructed the species phylogenies using a maximum likelihood supermatrix approach in IQ-TREE v.2.2.0 (Minh *et al.*, 2020). Node support/informativeness was calculated using 1000 bootstrap (BS) replicates and site concordance factors (sCF; Lanfear and Hahn, 2024). Following Hendriks *et al.* (2023), all phylogenies were calibrated using treePL (Smith and O’Meara, 2012) with the *Dressiantha bicarpellata* fossil (estimated at 93.6–89.3 Myr old; Gandolfo *et al.*, 1998) at the crown of order Brassicales. Node age ranges were calculated by running treePL for all 1000 BS trees, after which results were summarized in TreeAnnotator v.2.7.6 (Bouckaert *et al.*, 2014). To be consistent, we applied the same methods to all three genomes. Finally, phylogenies were visualized using R package ‘ggtree’ (Xu *et al.*, 2022), and tanglegrams of pairs of final species phylogenies were drawn using ‘phytools’ (Revell, 2012).

## RESULTS

### Genetic variation

**Fig. 1.**
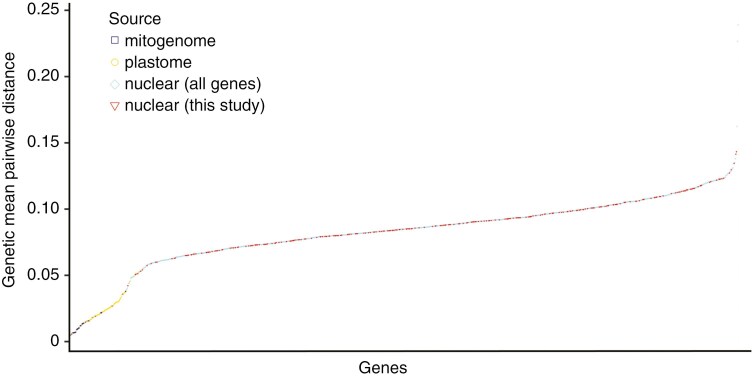
Genetic mean pairwise distance (MPD) across all studied genes. The colours represent the source genome: purple for mitochondrial (mitogenome), yellow for chloroplast (plastome), and blue (all genes) and red (genes used in this study) for nuclear genome. Genetic variation is lowest in mitochondrial genes, highest in nuclear genes, and intermediate in plastid genes.

Genetic variation was lowest in the mitogenome, followed by the plastome and the nuclear genome (Fig. 1), confirming our initial assumption. Mitogenomic gene MPD ranged from 0.5 % (*rps7* gene) to 3.8 % (*rps12* gene), with a mean of 1.4 % across all 32 genes studied (‘relaxed’ strategy; Supplementary Data Table S11). For the plastome, these values ranged from 0.4 % (*rps7* gene) to 5.9 % (*rps16* gene), with a mean of 2.7 % across all 76 genes studied (Supplementary Data Table S12). Finally, for the nuclear genome, values ranged from 3.9 % (B764-derived gene 2G38280) to 23.9 % (A353-derived gene 6559), with a mean of 8.8 % across all 1044 successfully aligned genes (8.9 % across the subset of 281 genes used in reconstructing the new nuclear phylogeny in this study; Supplementary Data Table S13).

### Global mitogenomic Brassicaceae phylogeny

**Fig. 2.**
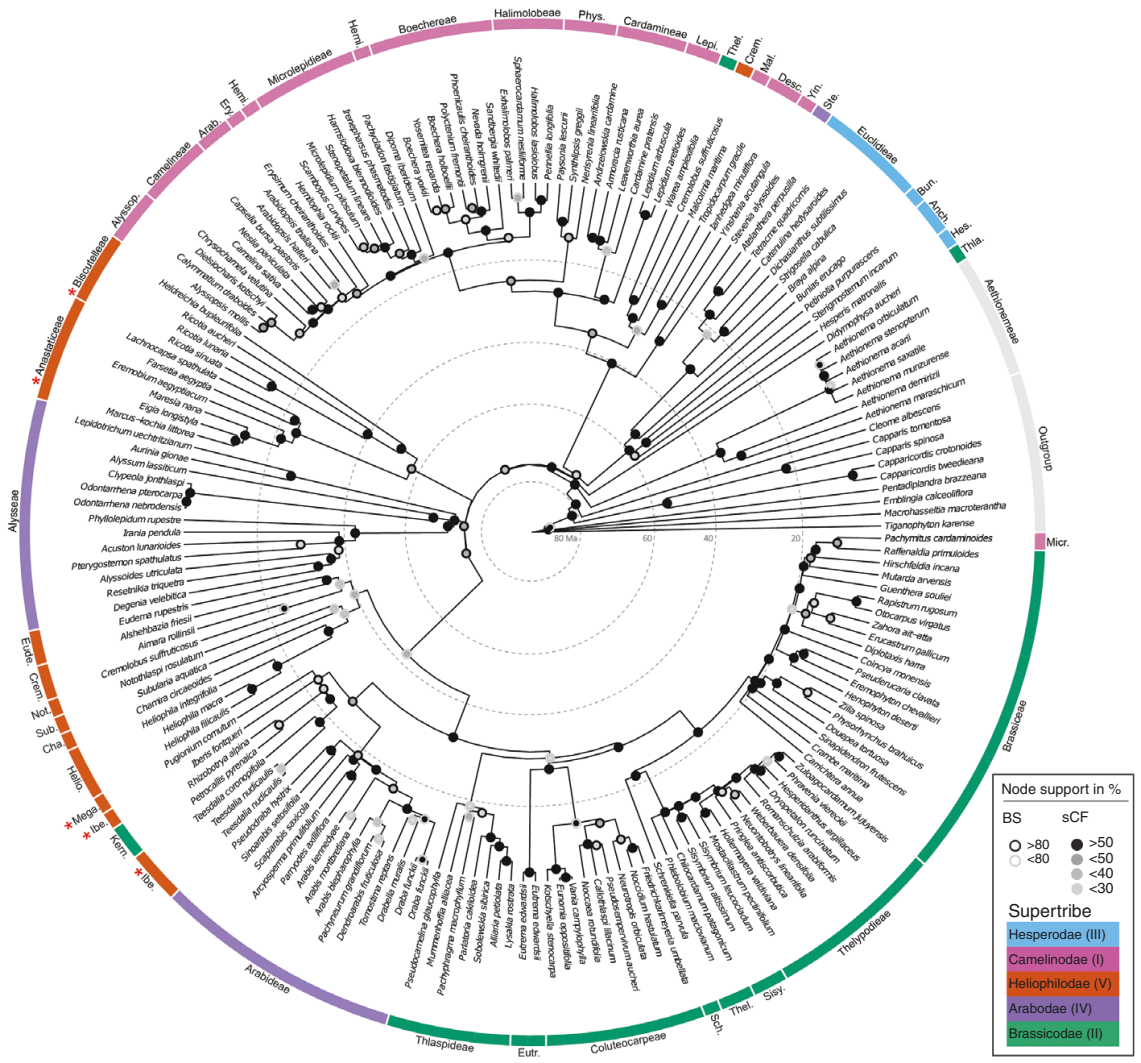
Time-calibrated genus-level mitogenomic Brassicaceae phylogeny based on the ‘selective’ strategy, from a maximum likelihood approach analysis of 32 mitochondrial genetic markers. Node support is indicated by circles, with outer circles representing bootstrap (BS) values and inner circles representing site concordance factors (sCF). Supertribes are colour-coded, and red asterisks mark rogue taxa (as defined by German *et al.*, 2023; Hendriks *et al.*, 2023; see also Table 2). Tribe names are given along the outer edge of the phylogeny. For a richly annoated and detailed version, see Supplementary Data Fig. S2. Abbreviations of tribes are as follows: Anch., Anchonieae; Arab., Arabidopsideae; Bun., Buniadeae; Cha., Chamireae; Crem., Cremolobeae; Desc., Descurainieae; Ery., Erysimeae; Eude., Eudemeae; Eutr., Eutremeae; Helio., Heliophileae; Hemi., Hemilophieae; Hes., Hesperideae; Ibe., Iberideae; Lepi., Lepidieae; Mal., Malcolmieae; Mega., Megacarpaeeae; Micr., Microlepidieae; Not., Notothlaspideae; Phys., Physarieae; Ste., Stevenieae; Sub., Subularieae; Thel., Thelypodieae; Thla., Thlaspideae; Yin., Yinshanieae.

We generated three novel mitogenomic Brassicaceae phylogenies based on ever-stricter sampling, thereby improving data density, at the cost of a reduction in species (and, as a result, genera and tribes) included (Table 1). Topologies from all three mitogenomic strategies (‘relaxed’, ‘selective’ and ‘stringent’) are highly similar, with the same backbone topology and general topological position of the tribes, indicating that the overall pattern in the mitogenomic phylogeny is not very sensitive to data selection (Supplementary Data Figs S1–S3). Unless stated otherwise, details and numbers are given for results from the ‘selective’ strategy (Fig. 2; Supplementary Data Fig. S2), because it offers the most balanced option between species and gene sampling. This mitogenomic sampling strategy resulted in a representation of 167 species, 145 genera (~40 % of the family) and 40 tribes (69 % of the family).

In line with previous findings (e.g. Nikolov *et al.*, 2019; Walden *et al.*, 2020; Hendriks *et al.*, 2023), we recovered the Brassicaceae family as a well-supported monophyletic group sister to family Cleomaceae (node support BS/sCF: 98/63.8; reported in this order in the remaining Results), with subfamily Aethionemoideae strongly supported as sister to subfamily Brassicoideae (the ‘core Brassicaceae’; 98/88.3). Surprisingly, we recovered a crown age of 77.0 Mya for the ‘selective’ strategy [95 % highest posterior density (HPD): 84.0–64.6 Mya; Supplementary Data Table S14], much older than any previous estimate (see Discussion). For the ‘relaxed’ and ‘stringent’ strategies, the crown age of the family was 75.5 Mya (HPD: 77.2–69.8 Mya) and 54.9 Mya (HPD: 60.0–46.7 Mya), respectively. In all three mitogenomic phylogenies, subfamily Brassicoideae was divided into two main clades, although this division was poorly supported (93/36.4), with extremely short branch lengths. The first of these main Brassicoideae clades was composed of supertribes Hesperodae (lineage III) and Camelinodae (lineage I) as poorly supported sisters (84/24.7), each non-monophyletic. The second Brassicoideae clade was a mix of subclades from supertribes Arabodae (lineage IV), Heliophilodae (lineage V) and Brassicodae (lineage II), with only the last a monophyletic group (except for two outliers; see ‘Rogue taxa and phylogenomic outliers’ below). Focusing on more recent clades, we found nearly all tribes (for exceptions, see ‘Rogue taxa and phylogenomic outliers’ below) recovered as strongly supported monophyletic groups (BS > 95 and sCF 50–80), supporting the taxonomic usage of the tribe as a valid taxonomic entity.

### Updated nuclear and plastome Brassicaceae phylogenies

**Fig. 3.**
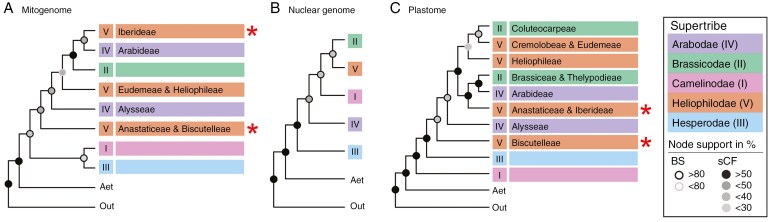
Comparison of Brassicaceae family-level phylogenetic topologies based on: (A) mitogenome (‘selective’ strategy); (B) nuclear genome; and (C) plastome sequence data. Tip label colours correspond to supertribes, with prime constituent tribes listed. Supertribal numbers follow those of Nikolov *et al.* (2019). Abbreviations: Aet., tribe Aethionemeae; Out., outgroup families. Rogue tribes are marked with a red asterisk (*).

In addition to our new global mitogenomic Brassicaceae phylogenies, we revisited the data of Hendriks *et al.* (2023) to generate updated nuclear and plastome Brassicaceae phylogenies using the same methodology as applied in our mitogenomic analysis, thereby avoiding methodologically derived discrepancies among the three datasets (Fig. 3). In our updated nuclear phylogeny (Supplementary Data Fig. S4), we recovered subfamilies Aethionemoideae and Brassicoideae as well-supported sisters (100/72.8) and all supertribes as defined by German *et al.* (2023) as monophyletic groups, with the main topology (‘backbone’) in line with that of the nuclear phylogeny of Hendriks *et al.* (2023). The crown age of the family was 39.5 Mya (HPD: 40.6–39.2 Mya), again older than the 24.5 Mya found by Hendriks *et al.* (2023) based on the same nuclear genomic dataset.

The main topology of our updated plastome phylogeny (Supplementary Data Fig. S5) is also in line with that of the plastome phylogeny of Hendriks *et al.* (2023), again with subfamilies Aethionemoideae and Brassicoideae moderately supported sisters (100/52.5) and only supertribes Camelinodae (sister to the rest of the Brassicoideae; 100/73.6) and Hesperodae (again sister to the rest of the Brassicoideae; 100/65.8) recovered as well-supported monophyletic groups (but with some outliers; see ‘Rogue taxa and phylogenomic outliers’ below). The crown age of the family was 35.7 Mya (HPD: 39.0–31.7 Mya) and therefore very close to that based on the nuclear genome, but again much older than the 20.2 Mya found from plastome data by Hendriks *et al.* (2023). In line with the plastome phylogeny of Hendriks *et al.* (2023), several supertribes were polyphyletic: Arabodae (tribe Alysseae not the direct sister to tribe Arabideae, as in the nuclear phylogeny); Brassicodae (tribes Brassiceae, Sisymbrieae and Thelypodieae sisters within this supertribe, but tribes Coluteocarpeae and Conringieae within supertribe Heliophilodae); and Heliophilodae (some tribes sister to tribes from supertribe Arabodae or Brassicodae; for results of rogue Heliophilodae taxa, see ‘Rogue taxa and phylogenomic outliers’ below). In nearly all these deep-node cases, support was (very) low (sCF generally < 50) and branch lengths short, suggesting strong conflict in the data. Importantly, as in the mitogenomic phylogeny, we recovered nearly all tribes as well-supported monophyletic groups in the nuclear and plastome phylogenies (BS > 95 and sCF 50–80; Supplementary Data Figs S1–S3), again confirming the delimitation of most tribes as valid taxonomic entities (for exceptions, see ‘Rogue taxa and phylogenomic outliers’ below).

### Rogue taxa and phylogenomic outliers

**Fig. 4.**
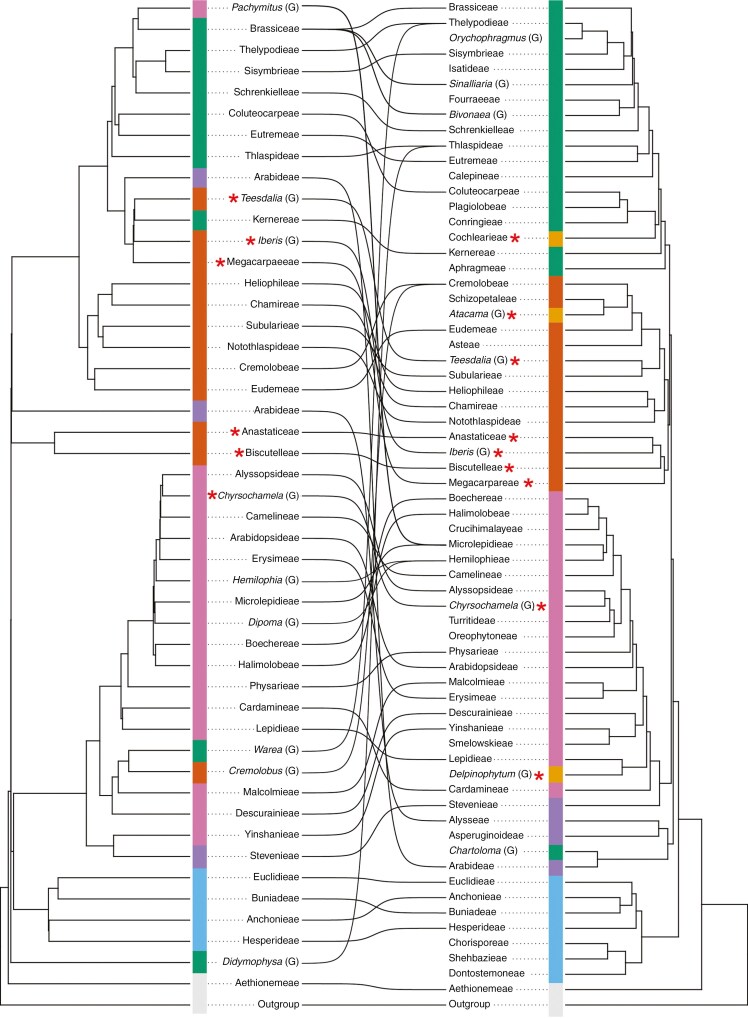
Tanglegram comparing Brassicaceae tribe-level phylogenies based on mitogenome (left) and nuclear genome (right) data. Colours represent supertribes as in Fig. 1, and red asterisks mark rogue taxa. All genera (labelled ‘G’) recovered as polyphyletic to their respective tribe are included. Note that more tribes were included in the nuclear phylogeny owing to higher data recovery; taxa without connecting lines indicate those missing from the mitogenomic phylogeny.

**Fig. 5.**
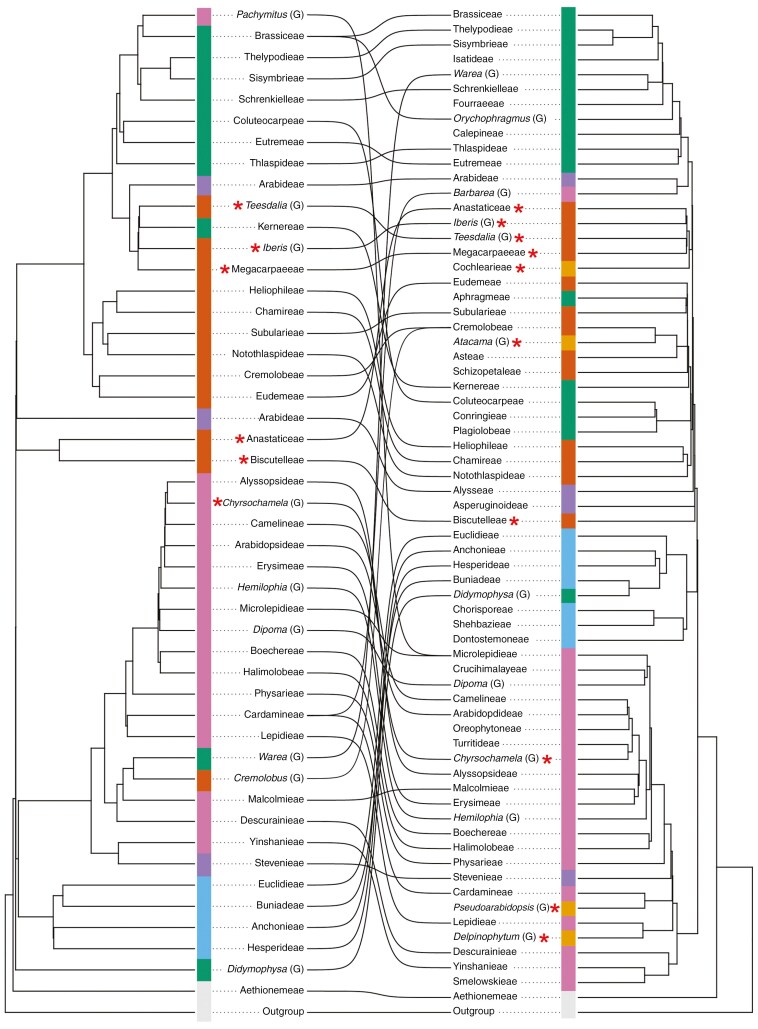
Tanglegram comparing Brassicaceae tribe-level phylogenies based on mitogenome (left) and plastome (right) data. Colours represent supertribes as in Fig. 1, and red asterisks mark rogue taxa. All genera (labelled ‘G’) recovered as polyphyletic to their respective tribe are included. Note that more tribes were included in the plastome phylogeny owing to higher data recovery; taxa without connecting lines indicate those missing from the mitogenomic phylogeny.

Cytonuclear discordance in the Brassicaceae based on plastome and nuclear genome sequence data has been well documented, and our results corroborate previous findings (Supplementary Data Fig. S6). We find cytonuclear discordance to be equally strong when comparing the mitogenomic results against the nuclear genomic results (Fig. 4). Surprisingly, our data also show strong discordance between the two (generally) maternally inherited organellar genomes, i.e. the mitogenome and the plastome (Fig. 5), a phenomenon we coin ‘mitoplastomic discordance’.

Although monophyly and strong support for the tribes within Brassicaceae were observed consistently across the phylogenomic analyses in this study, the placement of several tribes remained problematic (Table 2), echoing the findings of Nikolov *et al.* (2019) and Hendriks *et al.* (2023). Specifically, there was no consensus among the three genomic datasets to resolve unequivocally the positions of Anastaticeae, Biscutelleae, Iberideae and Megacarpaeeae (all belonging to supertribe Heliophilodae). Although a close relationship with tribe Heliophileae was evident, their relative positions varied across datasets, and these tribes frequently appeared sister to lineages from supertribes other than Heliophileae. For the previously unplaced tribe Cochlearieae (German *et al.*, 2023), the plastome phylogeny indicated a relationship with other rogue Heliophilodae tribes. In contrast, the nuclear phylogeny supported an association with tribes from supertribe Brassicodae; the latter relationship being significantly more robust based on node support.

**Table 2.** Brassicaceae family rogue taxa and phylogenomic outliers. Taxonomic classifications (first two columns) are based on tribe and supertribe assignments following German *et al.* (2023). Phylogenomic corroboration is scored as follows: ‘−’ (different tribe, different supertribe), ‘0’ (same tribe, different supertribe), and ‘+’ (same tribe, same supertribe). Likely phylogenomic outliers are indicated by an asterisk (*). Mitogenomic results from ‘selective’ strategy; when taxon only recovered in ‘relaxed’ strategy (when missing from ‘selective’ strategy) results are in square brackets.

Taxon	Tribe (supertribe)	Phylogenomic position						Comments
		Mitogenome		Plastome		Nuclear genome		
**Rogue tribes and previously unplaced taxa**								
Rogue tribe composed of *Cochlearia* and *Ionopsidium*	Cochlearieae (unplaced)	−	[Sister to *Teesdalia* (tribe Iberideae); with tribes Kernereae and Megacarpa each sister to tribe Arabideae]	−	Sister to clade of rogue tribes Anastaticeae, Iberideae and Megacarpaeeae, together within supertribe Heliophilodae	+	Sister to clade of tribes Coluteocarpeae, Conringieae and Plagiolobeae, within supertribe Brassicodae	Sister node support much higher in nuclear (sCF 96) than plastome (sCF 36.7) and very low (sCF 3) in mitogenomic phylogeny
Rogue tribe including *Diceratella*, *Farsetia*, *Lobularia* and *Parolinia*	Anastaticeae (Heliophilodae)	0	Tribe monophyletic and sister to tribe Biscutelleae, together making supertribe Heliophilodae polyphyletic	0	Tribe monophyletic and sister to *Iberis*, together sister to a clade including Cochlearieae, Megacarpaeeae and *Teesdalia*	+	Tribe monophyletic and sister to *Iberis*, together sister to tribe Biscutelleae	Most data confirm close relationship between tribes Anastaticeae and Biscutelleae, but the exact relationship remains unresolved
Rogue tribe including *Biscutella*, *Lunaria* and *Ricotia*	Biscutelleae (Heliophilodae)	0	Tribe monophyletic and sister to tribe Anastaticeae, together making supertribe Heliophilodae polyphyletic	−	Tribe monophyletic and sister to large clade of supertribes Arabodae, Brassicodae and remaining Heliophilodae	+	Tribe monophyletic and sister to clade of Anastaticeae and *Iberis*	
Rogue tribe composed of *Iberis* and *Teesdalia*	Iberideae (Heliophilodae)	−	*Iberis* sister to clade of *Teesdalia* and Kernereae	−	*Iberis* sister to Anastaticeae, with *Teesdalia* sister to tribe Megacarpaeeae	+	*Iberis* sister to Anastaticeae, with *Teesdalia* sister to *Subularia aquatica*	None of the genomes supports the combination of *Iberis* and *Teesdalia* into a single tribe, Iberideae
Rogue tribe composed of *Megacarpaea* and *Pugionium*	Megacarpaeeae (Heliophilodae)	−/?	*Pugionium* sister to clade of Iberideae and Kernereae (*Megacarpaea* not recovered)	−	Tribe monophyletic and sister to *Teesdalia*	+	Tribe monophyletic and sister to clade of Anastaticeae, Biscutelleae and *Iberis*	Close relationship to Iberideae follows from all three genomes
*Atacama nivea*	Unplaced (unplaced)	−	[Sister to *Didymophysa aucheri*, together sister to clade of CES tribes and tribe Heliophileae]	0	Sister to tribe Cremolobeae, within supertribe Heliophilodae	+	Sister to tribe Schizopetaleae, within supertribe Heliophilodae	Clearly associated with or within CES clade; nuclear results appear most solid
*Chrysochamela velutina*	Unplaced (Camelinodae)	0	Sister to tribe Alyssopsideae, together sister to tribe Camelineae, within supertribe Camelinodae	0	Sister to tribe Turritideae, together sister to clade including tribe Camelineae, within supertribe Camelinodae	+	Sister to tribe Alyssopsideae, together sister to tribe Turritideae, within supertribe Camelinodae	German *et al*. (2023) removed *Chrysochamela* from tribe Camelineae, which our results corroborate, however, our results suggest that it is closely related to Camelineae
*Delpinophytum patagonicum*	Unplaced (unplaced)	?	(Not recovered)	+	Sister to tribe Lepidieae, within supertribe Camelinodae	+	Sister to tribe Lepidieae, within supertribe Camelinodae	Available data confirm this species as sister to tribe Lepidieae
**Phylogenomic outlier taxa in one or more phylogenies in this study**								
*Aphragmus obscurus*	Aphragmeae (Brassicodae)	?	(Not recovered)	−	Within tribe Subularieae, within supertribe Heliophilodae*	+	Part of supertribe Brassicodae and sister to the rest of that supertribe	Position from plastome phylogeny likely false
*Barbarea vulgaris*	Cardamineae (Camelinodae)	?	(Not recovered)	−	Within tribe Arabideae, within supertribe Arabodae*	?	(Not recovered)	Raw data from Hendriks *et al*. (2023) did not pass the improved bioinformatic pipeline for mitogenome and nuclear genome analysis in the present study, suggesting poor-quality data for this sample
*Bivonaea lutea*	Brassiceae (Brassicodae)	0	[Within tribe Thelypodieae, within supertribe Brassicodae]	+	Sister to large clade containing most genera within tribe Brassiceae, within supertribe Brassicodae	0	Sister to tribe Fourraeeae, together sister to large clade containing tribes Isatideae, Sisymbrieae and Thelypodieae, within supertribe Brassicodae	Our results corroborate those of Hendriks *et al.* (2023), who also recovered *Bivonaea* well outside tribe Brassiceae
*Chartoloma platycarpum*	Isatideae (Brassicodae)	?	(Not recovered)	+	Within tribe Isatideae, within supertribe Brassicodae	−	Within genus *Draba*, within supertribe Arabodae*	Most nuclear data did not pass our improved bioinformatic pipeline, possibly explaining odd position
*Cremolobus suffruticosus* (two specimens)	Cremolobeae (Heliophilodae)	−/+	One specimen within supertribe Camelinodae*, the other sister to *Aimara rollinsii* in supertribe Heliophilodae	+	Both specimens in tribe Cremolobeae, within supertribe Heliophilodae	+	Both specimens in tribe Cremolobeae, within supertribe Heliophilodae	Two specimens from this species were included; unexpected position recovered for only one of these two
*Didymophysa aucheri*	Thlaspideae (Brassicodae)	−	Sister to subfamily Brassicoideae*	0	Sister to *Thlaspi arvense*, within tribe Thlaspideae, within supertribe Brassicodae	+	Sister to *D. fenestrata* in supertribe Brassicodeae	Position from mitogenomic phylogeny likely to be false
*Didymophysa fenestrata*	Thlaspideae (Brassicodae)	?	(Not recovered)	−	Within supertribe Hesperodae*	+	Sister to *D. aucheri* in supertribe Brassicodeae	Position from plastome phylogeny likely to be false
*Dipoma iberideum* and *Hemilophia rockii*	Hemilophieae (Camelinodae)	0	*Dipoma* sister to tribe Microlepidieae; *Hemilophia* sister to large clade of several tribes, all within supertribe Camelinodae	0	*Dipoma* sister to tribe Crucihimalayeae, together sister to tribe Microlepidieae; *Hemilophia* sister to large clade of several tribes; all within supertribe Camelinodae	+	Sister genera within tribe Hemilophieae, and together sister to tribe Microlepidieae, all within supertribe Camelinodae	The position of genus *Dipoma* might need revision based on the organellar genomes
*Macropodium nivale* and *Stevenia alyssoides*	Stevenieae (Arabodae)	−/?	*Stevenia alyssoides* within supertribe Camelinodae (also in ‘stringent’ strategy) (*Macropodium nivale* not recovered)	0	Together form tribe Stevenieae, within supertribe Camelinodae	0	Together form tribe Stevenieae, sister to supertribe Camelinodae	As in Walden *et al.* (2020), but in contrast to Hendriks *et al.* (2023), a close relationship to supertribe Camelinodae is inferred from all three genomes
*Orychophragmus violaceus*	Brassiceae (Brassicodae)	?	(Not recovered)	0	Sister to large clade containing tribes Brassiceae, Fourraeeae, Isatideae, Schrenkielleae, Sisymbrieae and Thelypodieae, within supertribe Brassicodae	0	Sister to genus *Raphanorhyncha*, within tribe Thelypodieae, within supertribe Brassicodae	Our results do not corroborate those of Hendriks *et al.* (2023), who recovered *Orychophragmus* as sister to the rest of tribe Brassiceae
*Pachymitus cardaminoides*	Microlepidieae (Camelinodae)	−	Sister to *Raffenaldia*, within supertribe Brassicodae*	+	Within tribe Microlepidieae, within supertribe Camelinodae	+	Within tribe Microlepidieae, within supertribe Camelinodae	Position from mitogenomic phylogeny likely to be false
*Kernera saxatilis, Petrocallis pyrenaica* and *Rhizobotrya alpina*	Kernereae (Brassicodae)	0	Within tribe Kernereae, but tribe within supertribe Heliophilodae (genus *Kernera* not recovered)	0	Within tribe Kernereae, but tribe sister to large clade of supertribe Heliophilodae representatives	+	Within tribe, within supertribe	Organellar genomes suggest relationship with supertribe Heliophilodae
*Pseudoarabidopsis toxophylla*	Unplaced (unplaced)	?	(not recovered)	−	Within tribe Cardamineae*	?	(Not recovered)	Position from plastome phylogeny likely to be false
*Sinalliaria limprichtiana*	Brassiceae (Brassicodae)	−	[Sister to Turritis, within supertribe Camelinodae]	+	Sister to rest of tribe Brassiceae, within supertribe Brassicodae	0	Sister to large clade of tribes Brassiceae, Isatideae, Sisymbrieae and Thelypodieae, within supertribe Brassicodae	Our results do not corroborate those of Hendriks *et al.* (2023), who recovered *Sinalliaria* within tribe Brassiceae
*Warea amplexifolia*	Thelypodieae (Brassicodae)	−	Within supertribe Camelinodae (also in ‘stringent’ strategy)*	−	Sister to tribe Isatideae, not within tribe Thelypodieae, but within supertribe Brassicodae	+	Within tribe Thelypodieae, within supertribe Brassicodae	Position from mitogenomic phylogeny likely to be false

Our analyses shed light on the placement of three previously unplaced monospecific genera (Table 2). Both nuclear and plastome phylogenies associated *Atacama* with other genera in the South American CES clade (composed of tribes Cremolobeae, Eutremeae and Schizopetaleae) within supertribe Heliophilodae. In addition, both phylogenies positioned the Argentinian endemic *Delpinophytum* as a sister to the large radiation of the genus *Lepidium* within supertribe Camelinodae. For *Chrysochamela*, the nuclear and mitogenomic analyses recovered the genus as sister to tribe Alyssopsideae, whereas plastome data suggested a sister relationship with tribe Turritideae. However, there is consensus among the results from all three genomes that *Chrysochamela* should be placed within supertribe Camelinodae.

We identified 15 ‘phylogenomic outliers’; specimens or species recovered in unexpected or doubtful positions in at least one of our three phylogenies (Table 2). In six cases (tribes Hemilophieae, Kernereae and Stevenieae, in addition to the genera *Bivonaea*, *Orychophragmus* and *Sinalliaria*), topological positions might have taxonomic significance and warrant further investigation (see Discussion). In contrast, the remaining nine outliers (representatives of *Aphragmus*, *Barbarea*, *Chartoloma*, *Cremolobus*, *Didymophysa*, *Pachymitus*, *Pseudoarabidopsis* and *Warea*) show clear topological mismatching in one of the datasets, appearing well outside their expected clades (see Discussion).

### *Phylogenomic placement of* Arabidopsis

Our sampling included the scientifically important model species *Arabidopsis thaliana* and *Arabidopsis halleri* (tribe Arabidopsideae), consistently recovered as sister species. However, phylogenomic discordance was evident in their topological placement: *Arabidopsis* was sister to tribes Alyssopsideae and Camelineae in the mitogenomic phylogeny (Fig. 2); to a larger clade including tribes Alyssopsideae, Boechereae, Camelineae, Microlepidieae and Physarieae in the nuclear phylogeny (Supplementary Data Fig. S4); and to tribes Camelineae, Crucihimalayeae, Hemilophieae and Microlepidieae in the plastome phylogeny (Supplementary Data Fig. S5).

## DISCUSSION

We present the most comprehensive mitogenomic phylogeny of the Brassicaceae to date, encompassing 167 species, 145 genera (representing 40 % of the family) and 40 tribes (69 % of the family; ‘selective’ strategy). We also reconstructed updated nuclear and plastome phylogenies to allow direct comparisons using the same raw sequencing data files (i.e. same specimens) and bioinformatic pipeline. Contrary to expectations, the slower-evolving mitogenome did not produce a well-supported phylogenetic backbone. Instead, we observed rampant cytonuclear and mitoplastomic discordance, particularly at the supertribal level, with each genomic dataset producing a different backbone topology. Nonetheless, our combined phylogenomic analyses support the taxonomic delimitation of 55 of 58 tribes (exceptions being tribes Iberideae, Hemilophieae and Subularieae), confirm the classification into two subfamilies (cf. German *et al.*, 2023) and shed more light on the evolutionary histories of several previously unplaced or taxonomically challenging lineages. Supertribal definitions as defined by German *et al.* (2023) are fully supported by our nuclear results, but only in part by the mitogenomic and plastomic results, suggesting a complex (early) evolutionary history of the family. Furthermore, our improved codon-aware gene alignments yielded significantly older crown age estimates for the Brassicaceae family (mitogenome ‘relaxed’ strategy: 75.5 Mya; ‘selective’ strategy: 77.0 Mya; ‘stringent’ strategy: 54.9 Mya; nuclear genome: 39.5 Mya; plastome: 35.7 Mya) in comparison to the 24.5 Mya estimate based on the nuclear genome reported by Hendriks *et al.* (2023). This highlights the strong sensitivity of calibration results to the choice of data and data handling, underscoring the urgent need for further research in this area.

### The backbone boggle

We aimed to reconstruct a robust Brassicaceae phylogenetic backbone based on conserved mitogenomic coding genes, a method shown to be effective in resolving deeper relationships in other plant families (Liu *et al.*, 2014; Rydin *et al.*, 2017; Soto Gomez *et al.*, 2020; Lin *et al.*, 2022; Xue *et al.*, 2022). Our analyses confirmed that mitochondrial gene exons have the lowest genetic variation among the three Brassicaceae genomes (Fig. 1). However, backbone topologies varied across the three genome phylogenies (Fig. 3), with none showing consistently higher node support across the deeper nodes (sCF ranges: mitogenome 24–88; nuclear 39–89; plastome 35–83). This suggests that substitution saturation and compositional heterogeneity are less significant than biological factors such as incomplete lineage sorting (Som, 2015), horizontal gene transfer (Filip and Skuza, 2021) and organelle capture (Stegemann *et al.*, 2012). Further research is necessary, because historical genome reshuffling presents a significant obstacle to resolving the Brassicaceae backbone fully (Degnan and Rosenberg, 2009), while at the same time, a hard polytomy (i.e. a rapid radiation following the origin of the Brassicaceae family) should be considered, as recently suggested for Fabaceae (Koenen *et al.*, 2021) and Poaceae (Grass Phylogeny Working Group III, 2025).

### Rampant mitoplastomic discordance

Surprisingly, we found strong incongruences between the mitogenome and plastome phylogenies (Fig. 5), a phenomenon we coin ‘mitoplastomic discordance’. Although differences between nuclear and organellar phylogenies (i.e. cytonuclear discordance) are common owing to distinct inheritance patterns (Larson *et al.*, 2024), it was expected that mitochondrial and plastid genomes would show strong phylogenetic concordance owing to: (1) their vertical transfer as (generally) non-recombinant genetic units; and (2) their joint uniparental inheritance (Birky, 2001; Tyszka *et al.*, 2023). On the contrary, recent studies have highlighted such mitoplastomic or ‘inter-organellar discordance’ before, for example in algae (Kao *et al.*, 2022), Rubiaceae (Rydin *et al.*, 2017), Orchidaceae (Li *et al.*, 2019) and *Potentilla* (Xue *et al.*, 2024). One explanation could be that maternal inheritance of mitochondria and plastids is not as strict as previously thought, and instances of both paternal and biparental inheritance in plants have been documented in both (Corriveau and Coleman, 1988; Shen *et al.*, 2015; Chybicki *et al.*, 2016; Van de Paer *et al.*, 2016). Additionally, lateral gene transfer (including organelle capture) can introduce foreign or chimeric sequences, leading to divergent evolutionary histories (Hao *et al.*, 2010; Rice *et al.*, 2013; Xian *et al.*, 2025).

### Taxonomic insights into rogue and unplaced taxa

In line with the work of Nikolov *et al.* (2019) and Hendriks *et al.* (2023), we faced several difficult-to-place taxa (mostly tribes; Table 2). These rogue taxa have the tendency to change position within the phylogeny, depending on the gene (sub)set analysed and the choice of phylogenetic inference tool (for further discussion, see Hendriks *et al.*, 2023) and are likely to be the result of inter-tribal or even inter-supertribal hybridizations (Hohmann *et al.*, 2015; Mandáková *et al.*, 2017*b*; Guo *et al.*, 2021; Huang *et al.*, 2023).

As in the study by Hendriks *et al.* (2023), we recovered the complex polyploid tribe Cochlearieae (Koch, 2012; Wolf *et al.*, 2021) as a monophyletic group sister to either a clade of tribes Coluteocarpeae, Conringieae and Plagiolobeae, within supertribe Brassicodae (nuclear phylogeny; sCF 96) or a clade of the rogue tribes Anastaticeae, Iberideae and Megacarpaeeae, within supertribe Heliophilodae (plastome phylogeny; sCF 37; conform Walden *et al.*, 2020). No representatives of this tribe were recovered in the ‘selective’ mitogenomic phylogeny, whereas results from the ‘relaxed’ mitogenomic phylogeny suggest a possible close relationship with *Teesdalia*. Notably, the nuclear phylogeny provided much stronger node support, highlighting higher topological variation in the plastome. Interestingly, Wolf *et al.* (2021) found no incongruences between the two ‘materal phylogenies’ when studying 19 *Cochlearia* taxa. This might suggest that mitoplastomic discordance is mostly an intertribal issue, but more research in other Brassicaceae tribes is needed. Finally, the changing phylogenetic placement of *Cochlearia* might be attributable to its hexaploid origin (Mandáková *et al.*, 2017*a*), which arose probably through hybridization between distantly related lineages (possibly from different tribes).

A close relationship among tribes Anastaticeae (mesotetraploid origin; Mandáková *et al.*, 2017*a*), Biscutelleae (mesopolyploid origin; Guo *et al.*, 2021), Iberideae (with at least *Iberis* of mesotetraploid origin; Mandáková *et al.*, 2017*a*) and Megacarpaeeae (polyploid origin; Yang *et al.*, 2020) was supported by all three genome phylogenies. Interestingly, all three genomes refute uniting *Iberis* and *Teesdalia* into a single tribe, as was traditionally accepted following the seminal work of Warwick *et al.* (2010). Instead, we found *Teesdalia* to be moderately supported (sCF 50) as a sister to tribe Kernereae (supertribe Brassicodae) in the mitogenomic phylogeny, moderately supported (sCF 55) as a sister to tribe Megacarpaeeae in the plastome phylogeny, and weakly supported (sCF 42) as a sister to *Subularia aquatica* (tribe Subularieae) in the nuclear phylogeny. At this point, the most practical solution to classify *Iberis* and *Teesdalia* is to establish two monogeneric tribes within supertribe Heliophilodae.

Our findings further unravel the intricate evolutionary relationships within the South American CES clade (composed of tribes Cremolobeae, Eudemeae and Schizopetaleae; Salariato *et al.*, 2016) and its close relatives, *Atacama nivea* and tribe Asteae. Each tribe was well supported as monophyletic in each of the three phylogenies, except for polyphyletic Cremolobeae in the plastome phylogeny (Supplementary Data Fig. S5). *Atacama nivea*, previously considered part of tribe Schizopetaleae but recently listed as unplaced (German *et al.*, 2023), grouped as sister to tribe Schizopetaleae in the nuclear phylogeny (sCF 48) and to Cremolobeae (sCF 63) in the plastome phylogeny and remains difficult to assign unequivocally to a tribe (cf. Toro-Núñez *et al.*, 2015). Likewise, tribe Asteae was sister to the CES clade in the nuclear phylogeny but nested within it in the plastome phylogeny. Tribe Subularieae, shifting affiliations across analyses and surprisingly recovered within the CES clade in the plastome phylogeny, is perhaps best treated as yet another ‘rogue tribe’, requiring further study. Moreover, in none of the phylogenetic results were its constituent genera, *Idahoa* and *Subularia*, recovered as sisters, meaning that the combination of these two genera within tribe Subularieae (*sensu*  German *et al.*, 2023) needs further study.

Likewise, our results further illuminate the phylogenetic position of two other enigmatic genera. First, we recovered the genus *Chrysochamela*, previously assigned to tribe Camelineae but recently reclassified as unplaced (German *et al.*, 2023), as sister to tribe Alyssopsideae in both mitogenomic and nuclear phylogenies. Second, aligning with the findings of Hendriks *et al.* (2023), we found the monospecific cushion-forming Argentinian endemic *Delpinophytum* [formerly placed in tribe Eudemeae (Salariato *et al.*, 2015*a*, b) but recently considered unplaced (German *et al.*, 2023)] as an early-diverging lineage sister to tribe Lepidieae. This relationship suggests that *Delpinophytum* might be best classified within tribe Lepidieae, as previously suggested by Al-Shehbaz *et al.* (2002).

### Taxonomic insights into phylogenomic outliers

We identified 15 ‘phylogenomic outliers’ that were retained for two reasons: (1) phylogenetic results from at least one genome align with prior findings, supporting the accuracy of specimen identification; and (2) we used raw sequencing data from the study by Hendriks *et al.* (2023), where these samples were phylogenetically recovered as sisters to their taxonomically expected relatives (based on the nuclear dataset). Given our strict thresholds for phylogenetic analysis (15 mitochondrial or five plastid genes, with plastid genes generally showing higher variation; Fig. 1), data deficiency cannot explain these outliers in most cases. Given these considerations and to avoid the risk of subjective data cherry-picking, we chose not to discard these results solely based on taxonomic expectations but to retain them for future study by the scientific community and give a detailed description here.

Importantly, in at least six cases, outliers might offer valuable taxonomic insights. For instance, the monospecific Mediterranean genus *Bivonaea*, previously assigned to different tribes (Warwick *et al.*, 2010; Koch, 2012) and most recently to tribe Brassiceae (German *et al.*, 2023), was retrieved as sister to tribe Fourraeeae in the nuclear phylogeny (cf. Hendriks *et al.*, 2023) and sister all other Brassiceae members (except *Sinalliaria*) in the plastome phylogeny. This suggests that its previous placement in the monogeneric tribe Bivonaeeae (cf. Koch, 2012) might be more practical until further study tells otherwise.

The Central Asian monospecific genus *Dipoma* was found to be sister to another Central Asian genus, *Hemilophia*, based on nuclear target capture results from Nikolov *et al.* (2019) and Hendriks *et al.* (2023), which led to its inclusion in the new tribe Hemilophieae by German *et al.* (2023). However, our mitogenomic results suggest a sister relationship of *Dipoma* to the Australian tribe Microlepidieae, whereas our plastome results suggest a sister relationship to yet another Central Asian tribe, Crucihimalayeae (cf. Walden *et al.*, 2020), together sister to tribe Microlepidieae. These results imply that the combination of *Dipoma* and *Hemilophia* into a single tribe might have been premature and that *Dipoma* deserves its own monospecific tribe.

We recovered tribe Stevenieae, composed of the closely related genera *Stevenia* and *Macropodium* (German *et al.*, 2009; German and Al-Shehbaz, 2010), as sister to the supertribe Camelinodae in both mitogenome and nuclear genome analyses, or placed within Camelinodae in the plastome phylogeny (or former lineage I, cf. Walden *et al.*, 2020). Therefore, inclusion of tribe Stevenieae in supertribe Arabodae by German *et al.* (2023) based on results from Hendriks *et al.* (2023) warrants revision. Interestingly, Stevenieae was recovered as the earliest diverging lineage of the respective supertribes in this study and Hendriks *et al.* (2023), a pattern that often signals historical inter-supertribal genetic admixture (e.g. hybridization; Funk, 1985). A similar case involves the polyploid genus *Orychophragmus* (tribe Brassiceae), which we recovered within tribe Thelypodieae in the nuclear genome phylogeny or as sister to a large clade of tribes (Brassiceae, Fourraeeae, Isatideae, Schrenkielleae, Sisymbrieae and Thelypodieae) in the plastome phylogeny, aligning broadly with the findings of Zhang *et al.* (2023). Although the genus shares conduplicate cotyledons with members of tribe Brassiceae, our results contradict its sister-group placement to that tribe as previously suggested (Hu *et al.*, 2016; Hendriks *et al.*, 2023).

For the small Central European tribe Kernereae (represented here by the monospecific diploid genera *Kernera*, *Petrocallis* and *Rhizobotrya*), both mitogenomic and plastome phylogenies suggest a close relationship with supertribe Heliophilodae rather than supertribe Arabodae (German *et al.*, 2023). Results from the mitogenome indicate a relationship with the European tribe Iberideae, whereas the plastome links it to the South American CES clade, closely resembling Koch’s (2012) finding of a relationship with tribe Buniadeae, the two together sister to a clade of several American tribes (Asteae, Cremolobeae and Schizopetaleae). Given the consistent clustering of the three genera of the tribe, data deficiency or methodological errors seem unlikely.

Lastly, the eastern China endemic monospecific genus *Sinalliaria* (tribe Brassiceae) was recovered in our nuclear phylogeny as sister to a clade comprising the tribes Brassiceae, Isatideae, Sisymbrieae and Thelypodieae, whereas in the plastome phylogeny, it was sister only to the remainder of tribe Brassiceae (cf. de Santana Lopes *et al.*, 2018; Hendriks *et al.*, 2023). Although its close relationship with tribe Brassiceae (supported also by shared conduplicate cotyledons) is evident, its precise phylogenetic placement requires further investigation.

## Conclusion

Our phylogenomic study is the first to compare all three plant genomes (nuclear, plastid and mitochondrial) at the family level in Brassicaceae, a family with a complex evolutionary history. Although determining the ‘best’ phylogeny remains challenging, the nuclear phylogeny is the most robust owing to its large dataset (281 genes filtered from a 1081 nuclear gene dataset), broad taxonomic sampling and strong alignment with historical morphology-based taxonomic classifications (von Hayek, 1911; Schulz, 1936; Janchen, 1942; Beilstein *et al.*, 2006; Al-Shehbaz, 2012). A key outcome of our tri-genomic approach is a near-universal support across all genomes for tribal circumscriptions *sensu*  German *et al.* (2023), emphasizing the validity and usefulness of the tribe as a taxonomic rank in Brassicaceae. Additionally, we confirm the monophyly of the two subfamilies [Aethionemoideae (comprising only monogeneric tribe Aethionemeae) and Brassicoideae (comprising the remaining 57 tribes)] and the early divergence of supertribes Hesperidae and Camelinodae within Brassicoideae. However, our family-wide mitogenomic phylogeny shows inconsistent supertribal relationships, and frequent shifts in rogue taxa (e.g. tribes Anastaticeae, Biscutelleae, Cochlearieae, Iberideae and Megacarpaeeae) among the three genome phylogenies remain, complicating our understanding of phylogenetic backbone. Until such conflicts are resolved, we advocate integration of the results from all three genomes to infer phylogenetic relationships and guide taxonomy.

Current phylogenomic methods relying on bifurcating trees cannot fully capture reticulate evolution, such as hybridization. Therefore, future studies should prioritize unravelling such complexities. Promising advances in Brassicaceae research, such as synteny analysis (Walden and Schranz, 2023) and ‘Parlog PhyloGenomics’ (Walden *et al.*, 2024), in addition to network-based methods, such as ‘PhyloNetworks’ (Solís-Lemus *et al.*, 2017), offer avenues to detect shared parentage in hybrid species. With increasing computational power, we anticipate that application of these algorithms to family-scale datasets will someday unveil the ‘true’ evolutionary history of the Brassicaceae.

## SUPPLEMENTARY DATA

Supplementary data are available at *Annals of Botany* online and consist of the following.

Table S1: sample data from the study by Hendriks *et al.* (2023) reanalysed for this study. Table S2: sample data from the study by Nikolov *et al.* (2019) reanalysed for this study. Table S3: previously published mitogenomes used to create mitogenomic target file. Table S4: mitogenomic genes used in this study. Table S5: mitogenomic target genes reference file. Table S6: previously published plastomes used to create plastome target file. Table S7: nuclear genome target genes reference file. Table S8: plastome target genes reference file. Table S9: chloroplast gene sampling overview. Table S10: nuclear genome gene sampling overview. Table S11: gene mean pairwise distance (MPD) for the mitogenomic genes (‘selective’ strategy). Table S12: gene mean pairwise distance (MPD) for the chloroplast genes. Table S13: gene mean pairwise distance (MPD) for the nuclear genes. Table S14: node age estimates (in millions of years ago with 95 % confidence interval). Figure S1: time-calibrated genus-level mitogenomic Brassicaceae phylogeny from ‘relaxed’ strategy. Figure S2: time-calibrated genus-level mitogenomic Brassicaceae phylogeny from ‘selective’ strategy with gene sampling heatmap. Figure S3: time-calibrated genus-level mitogenomic Brassicaceae phylogeny from ‘stringent’ strategy. Figure S4: time-calibrated genus-level nuclear Brassicaceae phylogeny. Figure S5: time-calibrated genus-level plastome Brassicaceae phylogeny. Figure S6: tanglegram comparing Brassicaceae tribe-level phylogenies based on plastome (left) and nuclear genome (right) data.

mcaf084_suppl_Supplementary_Tables_S1-S14

mcaf084_suppl_Supplementary_Figures_S1-S6

## Data Availability

Dominicus_et_al_2025_gene_alignments.zip: this file contains all gene alignments used to calculate genetic mean pairwise distances and to infer species phylogenies using IQ-TREE 2. The file can be downloaded from Zenodo via doi 10.5281/zenodo.14770522.

## References

[CIT0001] Al-Shehbaz IA. 2012. A generic and tribal synopsis of the Brassicaceae (Cruciferae). Taxon 61: 931–954.

[CIT0002] Al-Shehbaz IA, Mummenhoff K, Appel O. 2002. *Cardaria*, *Coronopus*, and *Stroganowia* are united with *Lepidium* (Brassicaceae). Novon 12: 5–11.

[CIT0003] Bailey CD, Koch MA, Mayer M, et al 2006. Toward a global phylogeny of the Brassicaceae. Molecular Biology and Evolution 23: 2142–2160. 16916944 10.1093/molbev/msl087

[CIT0004] Bakker FT, Culham A, Pankhurst CE, Gibby M. 2000. Mitochondrial and chloroplast DNA-based phylogeny of *Pelargonium* (Geraniaceae). American Journal of Botany 87: 727–734. 10811797

[CIT0005] Beilstein MA, Al-Shehbaz IA, Kellogg EA. 2006. Brassicaceae phylogeny and trichome evolution. American Journal of Botany 93: 607–619. 21646222 10.3732/ajb.93.4.607

[CIT0006] Beilstein MA, Al-Shehbaz IA, Mathews S, Kellogg EA. 2008. Brassicaceae phylogeny inferred from phytochrome A and *ndhF* sequence data: tribes and trichomes revisited. American Journal of Botany 95: 1307–1327. 21632335 10.3732/ajb.0800065

[CIT0007] Beilstein MA, Nagalingum NS, Clements MD, Manchester SR, Mathews S. 2010. Dated molecular phylogenies indicate a Miocene origin for *Arabidopsis thaliana*. Proceedings of the National Academy of Sciences of the United States of America 107: 18724–18728. 20921408 10.1073/pnas.0909766107 PMC2973009

[CIT0008] Birky CW. 2001. The inheritance of genes in mitochondria and chloroplasts: laws, mechanisms, and models. Annual Review of Genetics 35: 125–148. 10.1146/annurev.genet.35.102401.090231 11700280

[CIT0009] Bouckaert R, Heled J, Kühnert D, et al 2014. BEAST 2: a software platform for Bayesian evolutionary analysis. PLoS Computational Biology 10: e1003537. 24722319 10.1371/journal.pcbi.1003537 PMC3985171

[CIT0010] Chybicki IJ, Dering M, Iszkuło G, Meyza K, Suszka J. 2016. Relative strength of fine-scale spatial genetic structure in paternally vs biparentally inherited DNA in a dioecious plant depends on both sex proportions and pollen-to-seed dispersal ratio. Heredity 117: 449–459. 27577692 10.1038/hdy.2016.65 PMC5117843

[CIT0011] Corriveau JL, Coleman AW. 1988. Rapid Screening method to detect potential biparental inheritance of plastid DNA and results for over 200 Angiosperm species. American Journal of Botany 75: 1443–1458.

[CIT0012] Couvreur TLP, Franzke A, Al-Shehbaz IA, Bakker FT, Koch MA, Mummenhoff K. 2010. Molecular phylogenetics, temporal diversification, and principles of evolution in the mustard family (Brassicaceae). Molecular Biology and Evolution 27: 55–71. 19744998 10.1093/molbev/msp202

[CIT0013] Daniell H, Lin C-S, Yu M, Chang W-J. 2016. Chloroplast genomes: diversity, evolution, and applications in genetic engineering. Genome Biology 17: 134. 27339192 10.1186/s13059-016-1004-2 PMC4918201

[CIT0014] Degnan JH, Rosenberg NA. 2009. Gene tree discordance, phylogenetic inference and the multispecies coalescent. Trends in Ecology & Evolution 24: 332–340. 19307040 10.1016/j.tree.2009.01.009

[CIT0071] de Santana Lopes A, Gomes Pacheco T, do Nascimento Vieira L, et al 2018. The *Crambe abyssinica* plastome: Brassicaceae phylogenomic analysis, evolution of RNA editing sites, hotspot and microsatellite characterization of the tribe Brassiceae. Gene 671: 36–49. 29802993 10.1016/j.gene.2018.05.088

[CIT0015] Drouin G, Daoud H, Xia J. 2008. Relative rates of synonymous substitutions in the mitochondrial, chloroplast and nuclear genomes of seed plants. Molecular Phylogenetics and Evolution 49: 827–831. 18838124 10.1016/j.ympev.2008.09.009

[CIT0016] Duminil J. 2014. Mitochondrial genome and plant taxonomy. In: Besse P. ed. Molecular plant taxonomy: methods and protocols. Totowa, NJ: Humana Press, 121–140. 10.1007/978-1-62703-767-9_6 24415472

[CIT0017] Farhat P, Mandáková T, Divíšek J, Kudoh H, German DA, Lysak MA. 2023. The evolution of the hypotetraploid *Catolobus pendulus* genome — the poorly known sister species of *Capsella*. Frontiers in Plant Science 14: 1165140. 37223809 10.3389/fpls.2023.1165140 PMC10200890

[CIT0018] Filip E, Skuza L. 2021. Horizontal gene transfer involving chloroplasts. International Journal of Molecular Sciences 22: 4484. 33923118 10.3390/ijms22094484 PMC8123421

[CIT0019] Franzke A, German D, Al-Shehbaz IA, Mummenhoff K. 2009. *Arabidopsis* family ties: molecular phylogeny and age estimates in Brassicaceae. Taxon 58: 425–437.

[CIT0020] Franzke A, Lysak MA, Al-Shehbaz IA, Koch MA, Mummenhoff K. 2011. Cabbage family affairs: the evolutionary history of Brassicaceae. Trends in Plant Science 16: 108–116. 21177137 10.1016/j.tplants.2010.11.005

[CIT0021] Funk VA. 1985. Phylogenetic patterns and hybridization. Annals of the Missouri Botanical Garden 72: 681–715.

[CIT0022] Gandolfo M, Nixon K, Crepet W. 1998. A new fossil flower from the Turonian of New Jersey: *Dressiantha bicarpellata* gen. et sp. nov. (Capparales). American Journal of Botany 85: 964. 21684980

[CIT0023] German DA, Al-Shehbaz IA. 2010. Nomenclatural novelties in miscellaneous Asian Brassicaceae (Cruciferae). Nordic Journal of Botany 28: 646–651.

[CIT0024] German DA, Friesen N, Neuffer B, Al-Shehbaz IA, Hurka H. 2009. Contribution to ITS phylogeny of the Brassicaceae, with special reference to some Asian taxa. Plant Systematics and Evolution 283: 33–56.

[CIT0025] German DA, Hendriks KP, Koch MA, et al 2023. An updated classification of the Brassicaceae (Cruciferae). PhytoKeys 220: 127–144. 37251613 10.3897/phytokeys.220.97724 PMC10209616

[CIT0026] Givnish TJ. 2023. Plant biology: phylogenomics of mustards and their relatives. Current Biology: CB 33: R998–R1000. 37816328 10.1016/j.cub.2023.08.067

[CIT0027] Grass Phylogeny Working Group III. 2025. A nuclear phylogenomic tree of grasses (Poaceae) recovers current classification despite gene tree incongruence. New Phytologist 245: 818–834. 39568153 10.1111/nph.20263 PMC11655423

[CIT0028] Guo X, Liu J, Hao G, et al 2017. Plastome phylogeny and early diversification of Brassicaceae. BMC Genomics 18: 176. 28209119 10.1186/s12864-017-3555-3 PMC5312533

[CIT0029] Guo X, Mandáková T, Trachtová K, Özüdoğru B, Liu J, Lysak MA. 2021. Linked by ancestral bonds: multiple whole-genome duplications and reticulate evolution in a Brassicaceae tribe. Molecular Biology and Evolution 38: 1695–1714. 33331908 10.1093/molbev/msaa327 PMC8097306

[CIT0030] Hao W, Richardson AO, Zheng Y, Palmer JD. 2010. Gorgeous mosaic of mitochondrial genes created by horizontal transfer and gene conversion. Proceedings of the National Academy of Sciences of the United States of America 107: 21576–21581. 21115831 10.1073/pnas.1016295107 PMC3003057

[CIT0031] Hay NM, Windham MD, Mandáková T, et al 2023. A Hyb-Seq phylogeny of *Boechera* and related genera using a combination of Angiosperms353 and Brassicaceae-specific bait sets. American Journal of Botany 110: e16226. 37561651 10.1002/ajb2.16226

[CIT0034] Hendriks KP, Mandáková T, Hay NM, et al 2021. The best of both worlds: combining lineage-specific and universal bait sets in target-enrichment hybridization reactions. Applications in Plant Sciences 9: 1–12. doi: https://doi.org/10.1002/aps3.11438 PMC8312739 34336398

[CIT0033] Hendriks KP, Kiefer C, Al-Shehbaz IA, et al 2023. Global Brassicaceae phylogeny based on filtering of 1,000-gene dataset. Current Biology: CB 33: 4052–4068.e6. 37659415 10.1016/j.cub.2023.08.026

[CIT0035] Hohmann N, Wolf EM, Lysak MA, Koch MA. 2015. A time-calibrated road map of brassicaceae species radiation and evolutionary history. The Plant Cell 27: 2770–2784. 26410304 10.1105/tpc.15.00482 PMC4682323

[CIT0036] Hu H, Hu Q, Al-Shehbaz IA, et al 2016. Species delimitation and interspecific relationships of the genus *Orychophragmus* (Brassicaceae) inferred from whole chloroplast genomes. Frontiers in Plant Science 7: 1–10. 27999584 10.3389/fpls.2016.01826 PMC5138468

[CIT0039] Huang C-H, Sun R, Hu Y, et al 2016. Resolution of Brassicaceae phylogeny using nuclear genes uncovers nested radiations and supports convergent morphological evolution. Molecular Biology and Evolution 33: 394–412. 26516094 10.1093/molbev/msv226 PMC4866547

[CIT0037] Huang X-C, German DA, Koch MA. 2020. Temporal patterns of diversification in Brassicaceae demonstrate decoupling of rate shifts and mesopolyploidization events. Annals of Botany 125: 29–47. 31314080 10.1093/aob/mcz123 PMC6948214

[CIT0038] Huang Y, Guo X, Zhang K, Mandáková T, Cheng F, Lysak MA. 2023. The meso-octoploid *Heliophila variabilis* genome sheds a new light on the impact of polyploidization and diploidization on the diversity of the Cape flora. The Plant Journal 116: 446–466. 37428465 10.1111/tpj.16383

[CIT0040] Janchen E. 1942. Das System der Cruciferen. Österreichische Botanische Zeitschrift 91: 1–28.

[CIT0041] Johnson MG, Gardner EM, Liu Y, et al 2016. HybPiper: extracting coding sequence and introns for phylogenetics from high-throughput sequencing reads using target enrichment. Applications in Plant Sciences 4: apps.1600016. 27437175 10.3732/apps.1600016 PMC4948903

[CIT0042] Johnson MG, Pokorny L, Dodsworth S, et al 2019. A universal probe set for targeted sequencing of 353 nuclear genes from any flowering plant designed using k-medoids clustering. Systematic Biology 68: 594–606. 30535394 10.1093/sysbio/syy086 PMC6568016

[CIT0043] Kao T-T, Wang T-H, Ku C. 2022. Rampant nuclear–mitochondrial–plastid phylogenomic discordance in globally distributed calcifying microalgae. New Phytologist 235: 1394–1408. 35556250 10.1111/nph.18219 PMC9539906

[CIT0044] Kawabe A, Nukii H, Furihata HY. 2018. Exploring the history of chloroplast capture in *Arabis* using whole chloroplast genome sequencing. International Journal of Molecular Sciences 19: 602. 29463014 10.3390/ijms19020602 PMC5855824

[CIT0045] Koch MA. 2012. Mid-Miocene divergence of *Ionopsidium* and *Cochlearia* and its impact on the systematics and biogeography of the tribe Cochlearieae (Brassicaceae). Taxon 61: 76–92.

[CIT0046] Koenen EJM, Ojeda DI, Bakker FT, et al 2021. The origin of the legumes is a complex paleopolyploid phylogenomic tangle closely associated with the Cretaceous–Paleogene (K–Pg) mass extinction event. Systematic Biology 70: 508–526. 32483631 10.1093/sysbio/syaa041 PMC8048389

[CIT0047] Lanfear R, Hahn MW. 2024. The meaning and measure of concordance factors in phylogenomics. Molecular Biology and Evolution 41: msae214. 39418118 10.1093/molbev/msae214 PMC11532913

[CIT0048] Larson DA, Itgen M, Denton R, Hahn MW. 2024. Reconsidering cytonuclear discordance in the genomic age. EcoEvoRxiv. [preprint: not peer reviewed]. 10.1093/evolut/qpaf201 41055410

[CIT0049] Li Y-X, Li Z-H, Schuiteman A, et al 2019. Phylogenomics of Orchidaceae based on plastid and mitochondrial genomes. Molecular Phylogenetics and Evolution 139: 106540. 31252068 10.1016/j.ympev.2019.106540

[CIT0050] Lin Q, Braukmann TWA, Soto Gomez M, et al 2022. Mitochondrial genomic data are effective at placing mycoheterotrophic lineages in plant phylogeny. The New Phytologist 236: 1908–1921. 35731179 10.1111/nph.18335

[CIT0053] Liu L, Zhao B, Tan D, Wang J. 2011. Phylogenetic relationships of Brassicaceae in China: insights from a non-coding chloroplast, mitochondrial, and nuclear DNA data set. Biochemical Systematics and Ecology 39: 600–608.

[CIT0051] Liu Y, Cox CJ, Wang W, Goffinet B. 2014. Mitochondrial phylogenomics of early land plants: mitigating the effects of saturation, compositional heterogeneity, and codon-usage bias. Systematic Biology 63: 862–878. 25070972 10.1093/sysbio/syu049

[CIT0052] Liu L-M, Du X-Y, Guo C, Li D-Z. 2021. Resolving robust phylogenetic relationships of core Brassicaceae using genome skimming data. Journal of Systematics and Evolution 59: 442–453.

[CIT0054] Mandáková T, Li Z, Barker MS, Lysak MA. 2017a. Diverse genome organization following 13 independent mesopolyploid events in Brassicaceae contrasts with convergent patterns of gene retention. The Plant Journal: for Cell and Molecular Biology 91: 3–21. 28370611 10.1111/tpj.13553

[CIT0055] Mandáková T, Pouch M, Harmanová K, Zhan SH, Mayrose I, Lysak MA. 2017b. Multispeed genome diploidization and diversification after an ancient allopolyploidization. Molecular Ecology 26: 6445–6462. 29024107 10.1111/mec.14379

[CIT0056] Minh BQ, Schmidt HA, Chernomor O, et al 2020. IQ-TREE 2: new models and efficient methods for phylogenetic inference in the genomic era. Molecular Biology and Evolution 37: 1530–1534. 32011700 10.1093/molbev/msaa015 PMC7182206

[CIT0057] Nikolov LA, Shushkov P, Nevado B, et al 2019. Resolving the backbone of the Brassicaceae phylogeny for investigating trait diversity. The New Phytologist 222: 1638–1651. 30735246 10.1111/nph.15732

[CIT0058] Palmer JD. 1985. Comparative organization of chloroplast genomes. Annual Review of Genetics 19: 325–354. 10.1146/annurev.ge.19.120185.001545 3936406

[CIT0060] Palmer JD, Herbon LA. 1988. Plant mitochondrial DNA evolved rapidly in structure, but slowly in sequence. Journal of Molecular Evolution 28: 87–97. 3148746 10.1007/BF02143500

[CIT0059] Palmer JD, Adams KL, Cho Y, Parkinson CL, Qiu Y-L, Song K. 2000. Dynamic evolution of plant mitochondrial genomes: mobile genes and introns and highly variable mutation rates. Proceedings of the National Academy of Sciences of the United States of America 97: 6960–6966. 10860957 10.1073/pnas.97.13.6960 PMC34370

[CIT0061] Paradis E, Schliep K. 2019. ape 5.0: an environment for modern phylogenetics and evolutionary analyses in R. Bioinformatics 35: 526–528. 30016406 10.1093/bioinformatics/bty633

[CIT0062] Qiao J, Zhang X, Chen B, et al 2020. Comparison of the cytoplastic genomes by resequencing: insights into the genetic diversity and the phylogeny of the agriculturally important genus *Brassica*. BMC Genomics 21: 480. 32660507 10.1186/s12864-020-06889-0 PMC7359470

[CIT0063] Qiu Y-L, Lee J, Bernasconi-Quadroni F, et al 2000. Phylogeny of basal angiosperms: analyses of five genes from three genomes. International Journal of Plant Sciences 161: S3–S27.

[CIT0064] Ranwez V, Douzery EJP, Cambon C, Chantret N, Delsuc F. 2018. MACSE v2: toolkit for the alignment of coding sequences accounting for frameshifts and stop codons. Molecular Biology and Evolution 35: 2582–2584. 30165589 10.1093/molbev/msy159 PMC6188553

[CIT0065] Revell LJ. 2012. phytools: an R package for phylogenetic comparative biology (and other things). Methods in Ecology and Evolution 3: 217–223.

[CIT0066] Rice DW, Alverson AJ, Richardson AO, et al 2013. Horizontal transfer of entire genomes via mitochondrial fusion in the angiosperm *Amborella*. Science 342: 1468–1473. 24357311 10.1126/science.1246275

[CIT0067] Rydin C, Wikström N, Bremer B. 2017. Conflicting results from mitochondrial genomic data challenge current views of Rubiaceae phylogeny. American Journal of Botany 104: 1522–1532. 29885222 10.3732/ajb.1700255

[CIT0068] Salariato DL, Zuloaga FO, Al-Shebaz IA. 2015a. A taxonomic revision of the genus *Xerodraba* (Eudemeae, Brassicaceae). Phytotaxa 207: 39–67.

[CIT0069] Salariato DL, Zuloaga FO, Cano A, Al-Shehbaz IA. 2015b. Molecular phylogenetics of tribe Eudemeae (Brassicaceae) and implications for its morphology and distribution. Molecular Phylogenetics and Evolution 82 Pt A: 43–59. 25451804 10.1016/j.ympev.2014.09.030

[CIT0070] Salariato DL, Zuloaga FO, Franzke A, Mummenhoff K, Al-Shehbaz IA. 2016. Diversification patterns in the CES clade (Brassicaceae tribes Cremolobeae, Eudemeae, Schizopetaleae) in Andean South America. Botanical Journal of the Linnean Society 181: 543–566.

[CIT0072] Schranz ME, Mohammadin S, Edger PP. 2012. Ancient whole genome duplications, novelty and diversification: the WGD Radiation Lag-Time Model. Current Opinion in Plant Biology 15: 147–153. 22480429 10.1016/j.pbi.2012.03.011

[CIT0073] Schulz OE. 1936. Cruciferae. In: Engler A, Harms H. eds. Die natürlichen Pflanzenfamilien. Leipzig: Verlag von Wilhelm Englemann, 227–658.

[CIT0074] Shen J, Zhao J, Bartoszewski G, Malepszy S, Havey M, Chen J. 2015. Persistence and protection of mitochondrial DNA in the generative cell of cucumber is consistent with its paternal transmission. Plant and Cell Physiology 56: 2271–2282. 26412781 10.1093/pcp/pcv140

[CIT0075] Sloan DB, Alverson AJ, Chuckalovcak JP, et al 2012. Rapid evolution of enormous, multichromosomal genomes in flowering plant mitochondria with exceptionally high mutation rates. PLoS Biology 10: e1001241. 22272183 10.1371/journal.pbio.1001241 PMC3260318

[CIT0076] Smith SA, O’Meara BC. 2012. treePL: divergence time estimation using penalized likelihood for large phylogenies. Bioinformatics 28: 2689–2690. 22908216 10.1093/bioinformatics/bts492

[CIT0077] Solís-Lemus C, Bastide P, Ané C. 2017. PhyloNetworks: a package for phylogenetic networks. Molecular Biology and Evolution 34: 3292–3298. 28961984 10.1093/molbev/msx235

[CIT0078] Som A. 2015. Causes, consequences and solutions of phylogenetic incongruence. Briefings in Bioinformatics 16: 536–548. 24872401 10.1093/bib/bbu015

[CIT0079] Soto Gomez M, Lin Q, da Silva Leal E, et al 2020. A bi-organellar phylogenomic study of Pandanales: inference of higher-order relationships and unusual rate-variation patterns. Cladistics 36: 481–504. 34618964 10.1111/cla.12417

[CIT0080] Sousa F, Civáň P, Brazão J, Foster PG, Cox CJ. 2020. The mitochondrial phylogeny of land plants shows support for Setaphyta under composition-heterogeneous substitution models. PeerJ 8: e8995. 32377448 10.7717/peerj.8995 PMC7194085

[CIT0081] Stegemann S, Keuthe M, Greiner S, Bock R. 2012. Horizontal transfer of chloroplast genomes between plant species. Proceedings of the National Academy of Sciences of the United States of America 109: 2434–2438. 22308367 10.1073/pnas.1114076109 PMC3289295

[CIT0082] Tange O. 2011. GNU Parallel-the command-line power tool. Usenix Mag 36: 42.

[CIT0083] Toro-Núñez O, Al-Shehbaz IA, Mort ME. 2015. Phylogenetic study with nuclear and chloroplast data and ecological niche reveals *Atacama* (Brassicaceae), a new monotypic genus endemic from the Andes of the Atacama Desert, Chile. Plant Systematics and Evolution 301: 1377–1396.

[CIT0084] Tyszka AS, Bretz EC, Robertson HM, et al 2023. Characterizing conflict and congruence of molecular evolution across organellar genome sequences for phylogenetics in land plants. Frontiers in Plant Science 14: 1125107. 37063179 10.3389/fpls.2023.1125107 PMC10098128

[CIT0085] Unseld M, Marienfeld JR, Brandt P, Brennicke A. 1997. The mitochondrial genome of *Arabidopsis thaliana* contains 57 genes in 366,924 nucleotides. Nature Genetics 15: 57–61. 8988169 10.1038/ng0197-57

[CIT0086] Van de Paer C, Hong-Wa C, Jeziorski C, Besnard G. 2016. Mitogenomics of *Hesperelaea*, an extinct genus of Oleaceae. Gene 594: 197–202. 27601255 10.1016/j.gene.2016.09.007

[CIT0032] von Hayek A. 1911. Entwurf eines Cruciferen-Systems auf phylogenetischer Grundlage. Dresden: Heinrich.

[CIT0089] Walden N, Schranz ME. 2023. Synteny identifies reliable orthologs for phylogenomics and comparative genomics of the Brassicaceae. Genome Biology and Evolution 15: evad034. 36848527 10.1093/gbe/evad034 PMC10016055

[CIT0087] Walden N, German DA, Wolf EM, et al 2020. Nested whole-genome duplications coincide with diversification and high morphological disparity in Brassicaceae. Nature Communications 11: 3795. 10.1038/s41467-020-17605-7 PMC7393125 32732942

[CIT0088] Walden N, Kiefer C, Koch MA. 2024. Unravelling complex hybrid and polyploid evolutionary relationships using phylogenetic placement of paralogs from target enrichment data. *bioRxiv*: 2024.06.28.601132. [preprint: not peer reviewed]. 10.1093/sysbio/syag007 PMC13637605 41591393

[CIT0090] Warwick SI, Mummenhoff K, Sauder CA, Koch MA, Al-Shehbaz IA. 2010. Closing the gaps: phylogenetic relationships in the Brassicaceae based on DNA sequence data of nuclear ribosomal ITS region. Plant Systematics and Evolution 285: 209–232.

[CIT0091] Wolf E, Gaquerel E, Scharmann M, Yant L, Koch MA. 2021. Evolutionary footprints of a cold relic in a rapidly warming world. eLife 10: e71572. 34930524 10.7554/eLife.71572 PMC8741218

[CIT0092] Wolfe KH, Li WH, Sharp PM. 1987. Rates of nucleotide substitution vary greatly among plant mitochondrial, chloroplast, and nuclear DNAs. Proceedings of the National Academy of Sciences of the United States of America 84: 9054–9058. 3480529 10.1073/pnas.84.24.9054 PMC299690

[CIT0093] Xian W, Bao Z, Vorbrugg S, et al 2025. The structure of mitochondrial genomes is associated with geography in *Arabidopsis thaliana*. *bioRxiv*: 2025.01.11.632530. [preprint: not peer reviewed].

[CIT0094] Xu S, Li L, Luo X, et al 2022. ggtree: a serialized data object for visualization of a phylogenetic tree and annotation data. iMeta 1: e56. 38867905 10.1002/imt2.56 PMC10989815

[CIT0095] Xue J-Y, Dong S-S, Wang M-Q, et al 2022. Mitochondrial genes from 18 angiosperms fill sampling gaps for phylogenomic inferences of the early diversification of flowering plants. Journal of Systematics and Evolution 60: 773–788.

[CIT0096] Xue T-T, Janssens SB, Liu B-B, Yu S-X. 2024. Phylogenomic conflict analyses of the plastid and mitochondrial genomes via deep genome skimming highlight their independent evolutionary histories: A case study in the cinquefoil genus *Potentilla* sensu lato (Potentilleae, Rosaceae). Molecular Phylogenetics and Evolution 190: 107956. 37898296 10.1016/j.ympev.2023.107956

[CIT0097] Yang Q, Bi H, Yang W, et al 2020. The genome sequence of Alpine *Megacarpaea delavayi* identifies species-specific whole-genome duplication. Frontiers in Genetics 11: 1–9. 32849811 10.3389/fgene.2020.00812 PMC7416671

[CIT0098] Zhang K, Yang Y, Zhang X, et al 2023. The genome of *Orychophragmus violaceus* provides genomic insights into the evolution of Brassicaceae polyploidization and its distinct traits. Plant Communications 4: 100431. 36071668 10.1016/j.xplc.2022.100431 PMC10030322

